# Increased proinflammatory endothelial response to S100A8/A9 after preactivation through advanced glycation end products

**DOI:** 10.1186/1475-2840-5-6

**Published:** 2006-03-30

**Authors:** Philipp Ehlermann, Kai Eggers, Angelika Bierhaus, Patrick Most, Dieter Weichenhan, Johannes Greten, Peter P Nawroth, Hugo A Katus, Andrew Remppis

**Affiliations:** 1 Universität Heidelberg, Abteilung Innere Medizin III, Heidelberg, Germany; 2 Charité Campus Mitte, Kardiologie, Pneumologie und Angiologie, Berlin, Germany; 3 Universität Heidelberg, Abteilung Innere Medizin I, Heidelberg, Germany; 4 Instituto de Ciências Biomédicas Abel Salazar, Universidade do Porto, Portugal

## Abstract

**Background:**

Atherosclerosis is an inflammatory disease in which a perpetuated activation of NFkappaB via the RAGE (receptor for advanced glycation end products)-MAPK signalling pathway may play an important pathogenetic role. As recently S100 proteins have been identified as ligands of RAGE, we sought to determine the effects of the proinflammatory heterodimer of S100A8/S100A9 on the RAGE-NFkappaB mediated induction of proinflammatory gene expression.

**Methods:**

Human umbilical vein endothelial cells (HUVEC) were preincubated for 72 h with AGE-albumin or unmodified albumin for control, whereas AGE-albumin induction resulted in an upregulation of RAGE. Following this preactivation, cells were stimulated for 48 h with heterodimeric human recombinant S100A8/S100A9.

**Results:**

Heterodimeric S100A8/S100A9 enhanced secretion of IL-6, ICAM-1, VCAM-1 and MCP1 in AGE-albumin pretreated HUVEC in a dose dependent manner. These effects could not be detected after stimulation with the homodimeric proteins S100A8, S100A9, S100A1 and S100B. The effects of heterodimeric S100A8/S100A9 were reduced by inhibition of the MAP-kinase pathways ERK1/2 and p38 by PD 98059 and SB 203580, respectively.

**Conclusion:**

The heterodimeric S100A8/S100A9 might therefore play a hitherto unknown role in triggering atherosclerosis in diabetes and renal failure, pathophysiological entities associated with a high AGE burden. Thus, blocking heterodimeric S100A8/S100A9 might represent a novel therapeutic modality in treating atherosclerosis.

## Introduction

In recent years atherosclerosis has been identified as an inflammatory disease[1]. Animal experiments indicate that the RAGE (receptor of advanced glycation end products)/MAPK (mitogen activated protein-kinases)/NF-kappaB signal transduction pathway might play a critical role in perpetuating inflammatory responses[2]. RAGE is a member of the superfamily of immunoglobulin-like receptors and one of the receptors for AGEs[3]. After AGE binding to RAGE, NF-kappaB is activated via MAP-kinases, which in turn leads to an upregulation of several proatherogenic factors. In contrast to other signal transduction pathways RAGE induces a sustained activation of NF-kappaB[4]. The engagement of RAGE by AGE proteins may be considered as the 'first hit' in a two-stage model, in which the second phase of cellular perturbation is mediated by superimposed accumulation of modified lipoproteins, invading bacterial pathogens, ischemic stress and other factors[3].

Recently members of the EF-hand Ca^2+ ^binding protein family S100 were identified as ligands of RAGE[5,6]. At present 21 different S100 proteins are known[7], which are differentially expressed in several tissues exhibiting regulatory properties on a variety of cellular processes. Interestingly, in several metabolic, inflammatory and neoplastic diseases a differential expression of S100 proteins was observed, that is thought to play an important role in the pathogenesis of breast cancer, Alzheimer's disease, inflammatory bowel disease and transplant rejection[8]. Under the variety of S100 proteins S100A8 and S100A9 are expressed in neutrophils and monocytes[9] in which they represent a large proportion of the cytosolic protein[10]. S100A8/S100A9 are also known uneder a variety of alternative names like MRP-8/MRP-14, calprotectin, and calgranulin A/calgranulin B[8]. Activated neutrophils and monocytes play a central role in the initiation and progression of atherosclerosis. After activation of these cells S100A8 and S100A9 are released into the extracellular compartment via a tubulin dependent pathway[11], where they are known to promote the adhesion of neutrophils to endothelium, to act as chemotractants on monocytes and to enhance the uptake of LDL cholesterol by macrophages[12]. Moreover, elevated serum levels of S100A8 and S100A9 were reported in several inflammatory diseases like cystic fibrosis[13], inflammatory bowel disease[14], rheumatoid arthritis[15] and HIV infection[16].

Given these molecular findings we sought to determine whether proatherosclerotic and proinflammatory actions of S100A8 and S100A9 are involved in NF-kappaB dependent signalling.

## Materials and methods

### Materials

Human recombinant S100A8 and S100A9 was obtained from BMA Biomedicals (Switzerland). S100A8 and S100A9 are known to form heterodimers predominantly und physiological conditions[17]. The heterodimerization was performed by mixing both proteins in an equimolar solution containing 10mM Tris-HCl, pH 7.4, 0,1% sodium cholate, 1 mM EDTA, 1 mM 2-mercaptoethanol. Thereafter CaCl_2 _from a stock solution was added until a concentration of 2 mM and incubated under ambient temperature for 10 minutes. The efficiency of this procedure was assessed using a heterodimer specific ELISA (BMA, Switzerland). Endotoxin was removed using DetoxiGel^™ ^columns (Pierce, Rockford, IL, USA), which was confirmed by the E-Toxate assay (Sigma, St. Louis, MO, USA) employing limulus amebocyte lysate. Heterodimeric S100A8/S100A9 was stored in aliquots at -20°C. SB 203580 and PD 98059 were obtained from Calbiochem (CA, USA).

### Generation of AGEs

The generation of AGEs was performed as described[18]. Bovine serum albumin (100 g/l) was dissolved in phosphate buffered saline (pH 7.4) containing 0.5 M glucose, 1 mM PMSF, 2.5 mM EDTA, 100U/ml penicillin und 100 mg/l streptomycin and incubated under sterile conditions at 37°C for 6 weeks and then stored at -80°C. Mixtures of non-glycated bovine serum albumin and glucose of the same preparation were immediately frozen at -80°C and served as negative controls. These albumin-glucose solutions are referred to in the following as controls. The activity of AGE-preparations was confirmed using the one stage clotting assay as previously described[19], which is based on the induction of tissue factor on the surface of endothelial cells.

### Endothelial cell culture

Human umbilical cords were obtained from the department of obstetrics after informed consent in accordance with the ethical standards as formulated in the Helsinki declaration. Endothelial cells were harvested enzymatically as described[20] and were maintained in RPMI 1640 medium (Life Sciences, Germany) containing 20% fetal calf serum (Biochrom, Germany), 5 mM L-glutamine, 0.1 % Penicillin/Streptomycin, 25 mM HEPES, 50 U/ml heparin, 50 mg/l endothelial growth factor. For all experiments, cells of passage 3 were kept in RPMI 1640 medium containing 5% FCS for 24 h after confluence. Prestimulation of HUVEC was performed with a 1 mg/l concentration of AGE-preparations or albumin-glucose for controls. Except for the dose-response curves, S100 proteins were used for all experiments in an 1 μM (24 μg/ml heterodimeric S100A8/S100A9) concentration. Cell viability was assessed by exclusion of trypan blue and cell counting in a hemocytometer.

### Immunoblotting

Whole cell lysates were prepared by sonification of cell pellets in a buffer containing 10 mM HEPES pH 7.9, 10 mM KCL, 0,1 mM EGTA, 0,1 mM EDTA, 1 mM DTT, 0,5 mM PMSF. Protein concentration was determined using the DC Protein Assay (BioRad, Munich, Germany). Equal amounts (50 μg protein per lane) were resolved by 12 % SDS-PAGE using Novex 12% gels (Invitrogen, CA, USA) and transferred to PVDF membranes (Millipore, MA, USA). Immunoblots were developed using 1:2500 monoclonal mouse anti-RAGE (MAB5328 Chemicon, CA, USA) emloying the Western-Light PlusTM chemiluminescent detection kit (Tropix, Bedford, MA, USA).

### ELISA

ELISA kits for IL-6, ICAM-1 and VCAM-1 were purchased from Diaclone (Besancon, France), ELISA for MCP-1 from Biosource (CA, USA). All measurements were performed according to the manufacturer's guidelines.

### NF-kappaB ELISA

Both preparation of nuclear extracts by the mini extraction method and measurement of NF-kappa B on microtiter plates employing anti-NFκB c-Rel (Santa Cruz Biotechnology, CA, USA) was performed according to Schreiber[21].

### Statistical analysis

All values are expressed as mean ± standard deviation. Comparison between two groups was performed using the unpaired student T test employing the SPSS statistics software. A p-value below 0.05 was considered as significant.

## Results

**Figure 1:**
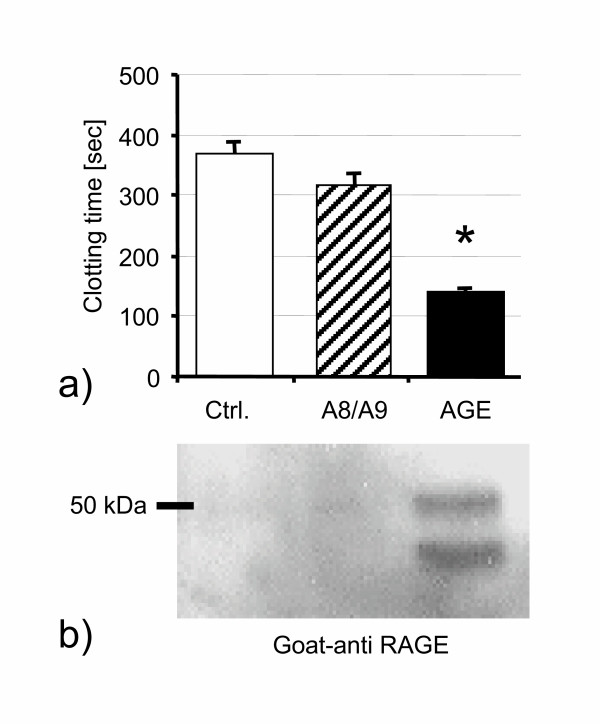
**a) **One stage clotting assay: AGE stimulation of HUVEC resulted in enhanced tissue factor expression indicated by a reduction of clotting time. Activation of the tissue factor pathway was not observed with S100A8/S100A9 alone. * p < 0.05 as compared to control and S100A8/S100A9. **b) **Immunoblot showing upregulation of RAGE upon AGE stimulation in HUVEC. S100A8/S100A9 had no effect, while costimulation by AGE and S100A8/S100A9 exhibited no additional effect (not shown). The doubled bands are probably due to degradation of RAGE.

As generation of AGEs is critically dependent to enviromental conditions like pH, protein concentration and various other unknown factors, we tested our AGE preparation for its functionality using the established *"one stage clotting assay"*, which is dependent on activation of tissue factor. Our AGE preparation significantly decreased clotting time indicating increased tissue factor activation (Fig. 1a). In contrast, the heterodimeric S100A8/S100A9 protein neither exhibited significant activation of clotting, nor did it show any effect on the expression of RAGE. AGE proteins, however, induced the expected upregulation of RAGE on HUVEC after 48 h of stimulation (Fig. 1b).

**Figure 2:**
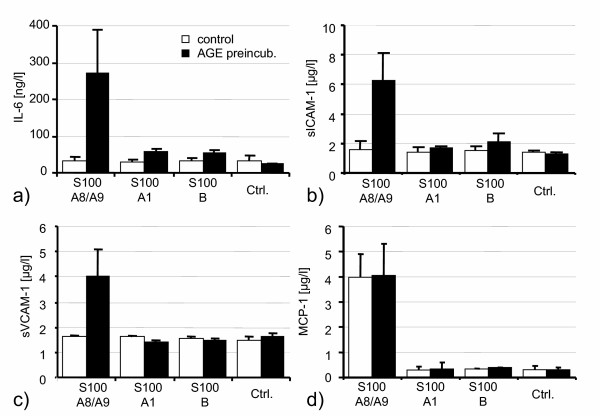
Stimulation of HUVEC by various S100 proteins for 48 h after 72 h preincubation with AGEs or albumin-glucose for control: Only after AGE pretreatment, 1 μM S100A8/S100A9 (24 μg/ml) induced a marked increase of IL-6 (**a**), sICAM-1 (**b**) and sVCAM-1 (**c**). MCP-1 (**d**) release was induced by S100A8/S100A9 irrespective of AGE pretreatment. 1 μM of S100A1 or S100B exhibited no effect.

**Figure 3:**
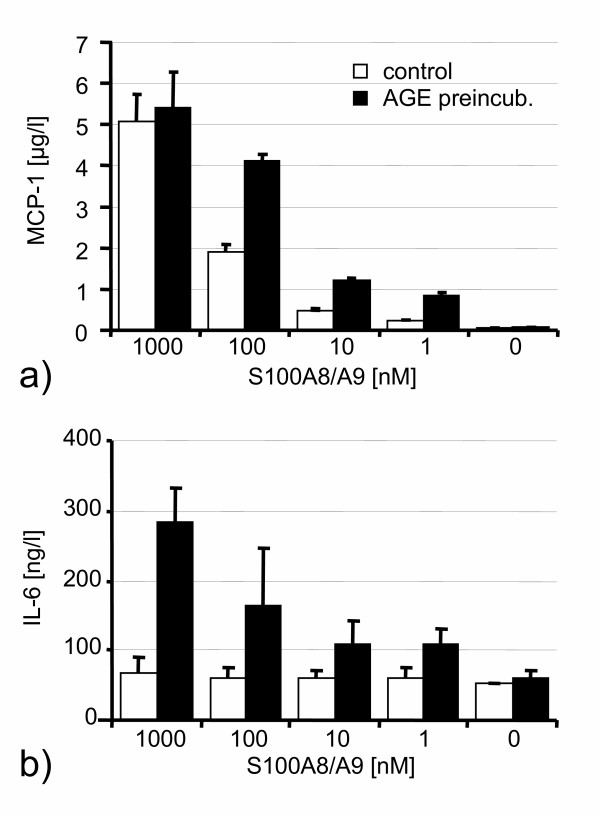
AGE pretreatment sensitized HUVEC for the stimulatory effects of S100A8/S100A9 regarding MCP-1 (**a**). Dose dependency of S100A8/S100A9 effects on IL-6 in AGE preincubated HUVEC (**b**). Similar results were obtained for sICAM-1 and sVCAM-1 (not shown).

HUVEC were stimulated with S100 proteins for 48 hours. Without AGE-preincubation, S100A8/S100A9 did not exhibit any effect on HUVEC regarding the release of IL-6, sICAM-1 and sVCAM-1. After preincubation with AGE proteins for 72 hours, however, stimulation with heterodimeric S100A8/S100A9 resulted in a marked increase of IL-6, sICAM-1 and sVCAM-1 measured in cell culture supernatants (Fig. 2a–c). These effects were not seen with two other members of the S100 family, S100A1 and S100B. Only MCP-1 release was significantly increased by S100A8/S100A9 irrespective of previous AGE stimulation (Fig. 2d). The dose-response curves for S100A8/S100A9 shows that AGE preincubation sensitizes HUVEC for the effects of lower concentrations of S100A8/S100A9 (Fig. 3a). In contrast, increasing concentrations of S100A8/S100A9 did not show any effect on IL-6 concentrations in controls, while AGE prestimulation resulted in a dose dependent effect of S100A8/S100A9 (Fig. 3b).

**Figure 4:**
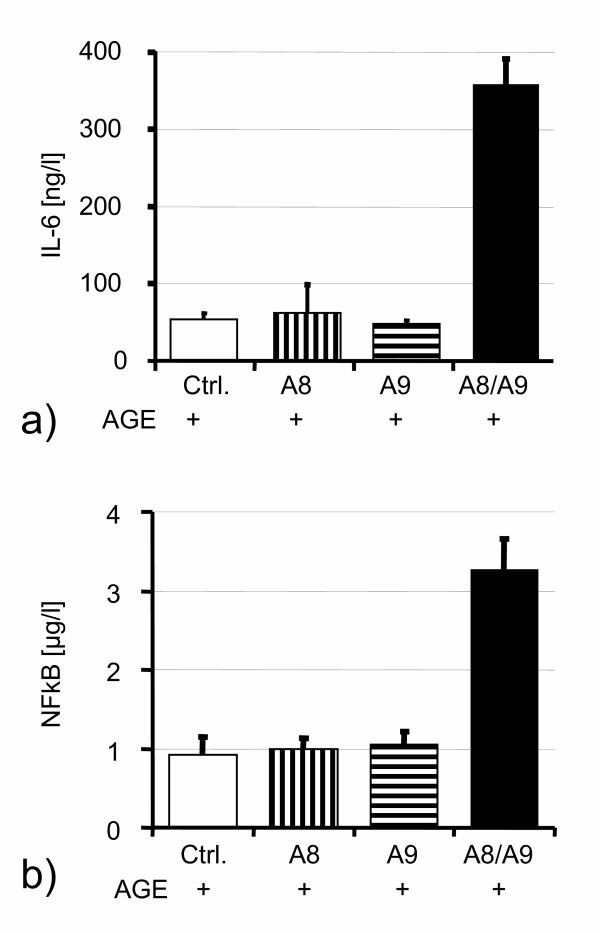
Stimulation of IL-6 (**a**) and NF-kappaB (**b**) by S100A8/S100A9 heterodimers but not with S100A8 or S100A9 homodimers. In controls, neither heterodimers nor homodimers were applied.

Heterodimeric S100A8/S100A9 was generated by coincubation of both recombinant proteins in the presence of 2 mM calcium. On these conditions heterodimers are rapidly formed, as confirmed by ELISA specific for the heterodimeric isoform (not shown). To confirm the specificity of the effects seen with heterodimeric S100A8/S100A9, recombinant homodimeric forms of S100A8 and S100A9 from the same preparation were used for differential stimulation experiments. While only the heterodimeric S100A8/S100A9 preparation showed effects on IL-6 release (Fig. 4a), no effects were seen for the respective homodimers. This was also the case for MCP-1, sICAM-1 and sVCAM-1 (not shown). Activation of the nuclear factor-kappa B (NF-kB) subunit cRel was only induced by the heterodimeric S100A8/S100A9 molecule (Fig. 4b), while both homodimers again showed no effect.

**Figure 5:**
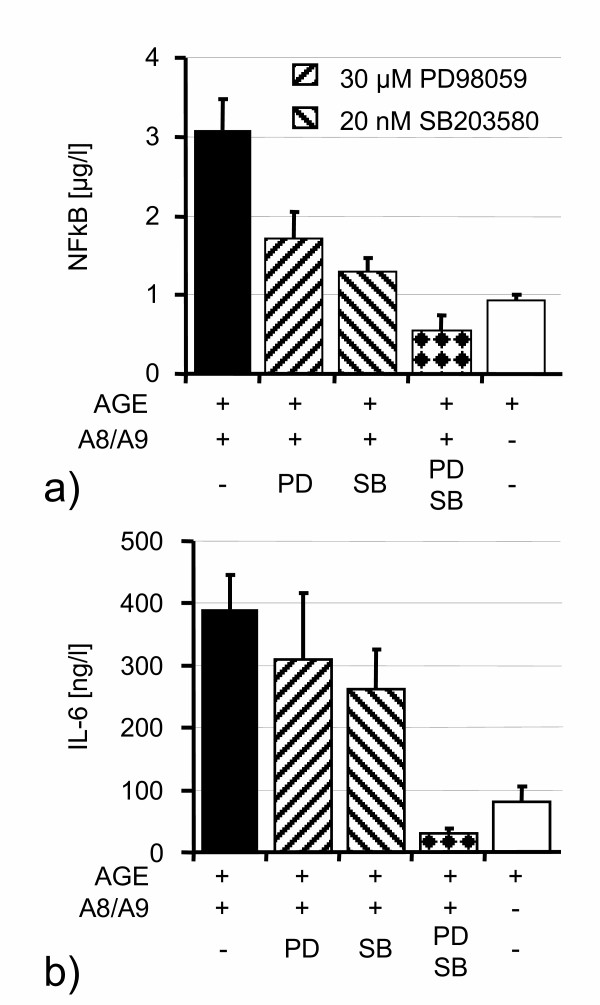
The proinflammatory effect of 1 μM heterodimeric S100A8/S100A9 on AGE pretreated HUVEC was reduced by blockers of the MAP-kinases Erk1/2 (PD98059) and p38 (SB 203580). Combined blocking of both MAP-kinases resulted in complete inhibition of NF-kappaB activation (**a**) and IL-6 release (**b**).

**Figure 6:**
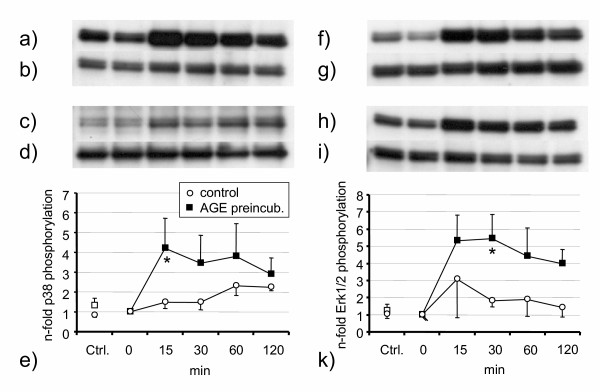
Stimulation of AGE-pretreated (black squares) HUVEC with heterodimeric S100A8/S100A9 resulted in phosphorylation of both MAP kinases p38 (**e**) and Erk1/2 (**k**) within 15 minutes. Representative immunoblots for AGE preincubated HUVEC are shown for phosphorylated p38 (**a**), total p38 (**b**), phosphorylated Erk1/2 (**f**) and total Erk1/2 (**g**). Control experiments: Phosphorylated p38 (**c**), total p38 (**d**), phosphorylated Erk1/2 (**h**) and total Erk1/2 (**i**) *p < 0.05 AGE pretreatment vs. albumin-glucose (open circles) for control.

In order to identify the involved MAP-kinase pathways, we performed blocking experiments with inhibitors of the Erk1/2 and p38 pathways, as these are known to be involved in RAGE dependent signalling[2]. Addition of only one of the two MAPK-inhibitors PD 98059 or SB 203580 resulted in a moderate decrease of IL-6 concentration or NF-kappaB activation after S100A8/S100A9 stimulation, whereas a combination of both inhibitors decreased proinflammatory activation even below control levels (Figure 5). These data strongly suggest that the observed effects are dependent on both MAP-kinase pathways and that blocking of only one of these pathways can be at least partially compensated by the other pathway. Because blocking experiments with kinase inhibitors can be influenced by unspecific effects of these agents, we used phospho-specific antibodies, which specifically detect the activated forms of Erk1/2 and p38. After AGE preincubation, stimulation of HUVEC with S100A8/S100A9 resulted in rapid activation of Erk1/2 and p38 within 15 minutes (Figure 6). Compared to controls without AGE preincubation, S100A8/S100A9 had no significant effects on MAP-kinase activation.

## Discussion

The activation of proinflammatory signalling pathways by RAGE is considered as one important pathogenetic feature in the initiation of atherosclerosis, especially in conditions in which RAGE-ligands accumulate such as in diabetes mellitus and chronic renal failure[22]. The engagement of RAGE by AGEs, a heterogeneous group of glycoxidated proteins, was proposed as a "first hit" in a two stage model of inflammation. Several factors are discussed to be involved in the "second hit", including oxidized lipoproteins and bacterial pathogens[3]. Since S100B and EN-RAGE (S100A12), proteins of the S100/calgranulin family, are known to interact with RAGE[5,6], the aim of our study was to evaluate, whether the proinflammatory S100 proteins S100A8 and S100A9, being expressed and secreted by mononuclear cells, would enhance activation of the RAGE/NF-kappaB axis thus providing a novel proatherogenetic mechanism.

### AGE proteins

The term AGEs describes a large group of heterogeneous molecules, which are formed by glycoxidation processes, depending on enviromental conditions[23,24]. Imitating the in vivo process, AGEs can be generated in-vitro by incubation of protein solutions with glucose under oxygen atmosphere at 37°C. These AGEs vary in their composition and molecular properties. Although this might be a disadvantage in contrast to purified single AGE compounds like N^epsilon^- carboxy-methyl-lysine (CML) from the biochemical view, this model employing a mixture of AGEs is much closer to pathophysiologial conditions found in vivo and observed in diabetes mellitus. The functional activity of the AGEs preparations used were confirmed by activated clotting induced in endothelial cells and augmented RAGE expression confirmed by Western blot (Fig. 1). Importantly, activation of clotting was neither seen with the potential RAGE ligands S100A8 and S100A9, nor with other S100 proteins.

### Preactivation of HUVEC by AGEs

For a better discrimination of AGE and S100 effects, the preincubation of HUVEC with AGEs was terminated after three days, when HUVEC monolayers were washed to remove AGEs. Furthermore, the AGE concentration (1 μg/l) used in our study was markedly lower as compared to other studies, in which up to 600 μg/l were used[25]. Consequently, no differences in proinflammatory activation were seen between AGE preincubated cells and albumin-glucose controls at baseline (data not shown). Upon induction with S100A8/S100A9, however, a marked difference was seen between AGE prestimulated and control cells, as only HUVEC sensitized by AGEs showed a significant proinflammatory response, evidenced by increased cytokine release, adhesion molecules, MAP kinase and NF-kappa B cRel activation. Interestingly, the only exception in our setting of experiments was MCP-1, which was released after S100A8/S100A9 stimulation irrespective of whether cells were preincubated with AGEs or not, implying that receptors different from RAGE and/or other AGE-binding sites are involved in S100A8/S100A9 dependent MCP-1 expression. However, dose response curves revealed, that AGE treated HUVEC exhibited a stronger inflammatory response to lower concentrations of S100A8/S100A9 with respect to MCP-1 release. As MCP-1 mediated activation and migration of monocytes plays a central role in the initiation of atherosclerosis[26] as well as in the development of restenosis after angioplasty[27], the proinflammatory stimulation of endothelial cells by heterodimeric S100A8/S100A9 protein may thus represent a hitherto unknown critical mechanism in the early propagation of atherosclerosis.

### Differential effects of hetero- and homodimers

Control experiments revealed that stimulation of HUVEC with various homodimeric S100 proteins (S100A1, S100B, S100A8, S100A9) does not result in any proinflammatory activation. These data not only underscore that proinflammatory effects of heterodimeric S100A8/S100A9 were specific in our experimental setting, but also add evidence to the specific physiological properties of this dimeric molecule. In acute and activated chronic inflammation the heterodimeric S100A8/S100A9 was found to be the predominant isoform[15], exhibiting molecular characteristics that are clearly distinguishable from both homodimeric isoforms[17]. In this context it has been speculated that either the heterodimeric protein forms an epitope, that specifically interacts with defined cellular receptors, or only the formation of heterodimeric S100A8/S100A9 would allow the bridging of two different epitopes[28,29]. Interestingly, Kerkhoff et al showed that only the heterodimer is able to bind arachidonic acid with high affinity resulting in a complex that interacts with CD36 facilitating fatty acid uptake by endothelial cells[30]. This finding sheds new light on the link between S100A8/S100A9 and RAGE, as recently RAGE overexpression was shown to be associated with an enhanced COX-2 expression in atherosclerotic plaques of diabetic patients[31].

### Potential receptors for S100A8/S100A9

As many authors employ soluble RAGE (sRAGE) to control for RAGE specific effects it might appear as a limitation, that we did not include any such experiments to our study. There is however ample evidence that control experiments using sRAGE certainly do not control for RAGE specific effects. They much more underscore the concept that RAGE ligands represent a group of multireceptor ligands whereas the employment of sRAGE simply sequesters those ligands thereby preventing their interaction with other receptors in addition to RAGE. Interestingly studies in RAGE-/- mice demonstrate that the protection conferred by RAGE deficiency is lower than that mediated by sRAGE. Moreover RAGE-/- mice can be protected by sRAGE in certain settings of adaptive immune response implying that abounding RAGE ligands may overwork the RAGE pathway and coactivate other receptors (for review see [32]) it is against this background that there still is a controversial debate as far as the endothelial receptors of S100A8/S100A9 are concerned. S100A8/S100A9 was reported to bind to heparan sulfate glycosaminoglycans on a human microvascular endothelial cell line (HMEC-1)[33], that neither expresses CD36 nor RAGE. In contrast, Hofmann et al showed that S100A12, which is a close homologue of S100A9, clearly binds to RAGE on HUVEC consequently activating a proinflammatory axis via NF-kappaB[5], while Srikrishna et al. [34] found that S100A8/S100A9 binds to carboxylated N-glycans, which are components of RAGE. It is therefore conceivable that heterodimeric S100A8/S100A9 is a ligand that binds to various cell receptors giving rise to a differentiated downstream signalling. The latter is further implied by the differential gene activation described in the present study, demonstrating that S100A8/S100A9 induces MCP-1 release in the absence of AGEs, while prestimulation with AGEs evokes S100A8/S100A9 mediated release of ICAM, VCAM and IL-6.

### Signalling pathways

RAGE dependent inflammatory processes mainly recruit MAP kinases p38 and Erk1/2[2] and a downstream activation of the transcription factor NF-kappaB[4] for induction of proinflammatory and procoagulatory gene expression. As demonstrated here, stimulation of HUVEC by S100A8/S100A9 critically depends on these two MAP kinases, since blocking of both pathways completely abolished the proinflammatory effects. In a recently published study using oligonucleotide microarray technology, stimulation of human microvascular endothelial cells with 200 μg/ml heterodimeric S100A8/S100A9 was shown to result in an upregulation of several genes that are known to promote platelet aggregation, inflammation, and endothelial permeability[35]. As cardiovascular events can be precipitated by acute respiratory infections[36], an increase of S100A8/S100A9 blood levels during acute infection in diabetic patients with increased RAGE expression and sustained NF-kappaB activation within atherosclerotic plaques might be an attractive molecular explanation for the excess morbidity and mortality in diabetes. Given these results, blocking of S100A8/S100A9 effects might thus represent a novel therapeutic strategy in the prevention or reversal of atherosclerotic complications especially in Diabetes mellitus or chronic renal failure.

## Abbreviations

AGE: Advanced glycation end products; HUVEC: Human umbilical vein endothelial cells; MAPK: Mitogen activated protein-kinases; RAGE: Receptor for advanced glycation end products

## Competing interests

The author(s) declare that they have no competing interests.

## Authors' contributions

PE, JG and AR were responsible for the preparation of the manuscript. Cell culture experiments were performed by PE and KE, protein purification and AGE generation by PE and DW, ELISA by PM, NF-kappaB Assay by KE and AB. PN, HK and AR conceived of the study, and participated in its design and coordination and helped to draft the manuscript. All authors read and approved the final manuscript.
